# Risk factors for recurrence of FMD outbreaks in Iran: a case-control study in a highly endemic area

**DOI:** 10.1186/s12917-018-1580-3

**Published:** 2018-08-29

**Authors:** Kayhan Ilbeigi, Saied Bokaie, Sina Aghasharif, Ricardo J. Soares Magalhães, Mohamad Rashtibaf

**Affiliations:** 1 Department of Clinical Sciences, Faculty of Veterinary Medicine, Islamic Azad University of Garmsar, Garmsar, Iran; 2 Department of Food Hygiene and Quality Control, Faculty of Veterinary Medicine, University of Tehran, Tehran, Iran; 3 UQ Spatial Epidemiology Laboratory, School of Veterinary Science, The University of Queensland, Gatton, Australia; 4 Children’s Health and Environment Program, Child Health Research Centre, The University of Queensland, Brisbane, Australia; 5 Iranian Veterinary Organization, Provincial Department of Surveillance and Disease Control, Khorasan Razavi, Islamic Republic of Iran

**Keywords:** Foot-and-mouth disease, Risk factors, Case-control study, Logistic regression analysis

## Abstract

**Background:**

Foot-and-mouth disease (FMD) is an acute viral disease of cloven-hoofed animals with high economic impact. FMD remains endemic in Iran particularly in the livestock-dense province of Khorasan Razavi in northeastern Iran where FMD outbreaks continuously occur. In this study, we aimed to quantify risk factors for the recurrence of FMD outbreaks in Iran by analyzing a time-series of FMD outbreak data from the province of Khorasan Razavi.

**Results:**

This study used FMD outbreak data collected from 2012 to 2014. Data were collected by local offices of the Iranian Animal Disease Department and the veterinarian of the veterinary council of the Khorasan Razavi province. An outbreak investigation questionnaire was delivered to 127 farms, including 46 case farms (FMD-infected) and 81 control farms (FMD-free). To quantify and compare the odds of exposure to a risk factor in FMD-infected farms versus FMD-free farms, logistic regression models were built using SPSS software version 16.

Our results of multivariable logistic regression indicate that hygienic status of the farm (OR = 11.83; CI = 3.38–41.43), FMD vaccination status (OR = 0.06; CI = 0.01–0.68), transportation of livestock (OR = 0.40; CI = 0.163–0.981) and inhibition of livestock dealers’ entry into the farm (OR = 0.36; CI = 0.12–1.09) were identified as important risk factors for farm-level FMD infection.

**Conclusion:**

This study generated much needed evidence on a set of modifiable risk factors for the recurrence of FMD outbreaks in the high risk province of Khorasan Razavi. This information can be used to improve existing national FMD control program and suggest new guidelines to prevent FMD outbreaks in the country.

## Background

Foot-and-mouth disease (FMD) is considered one of the highly contagious viral diseases affecting all cloven-hoofed animals. Among these animals, cattle are the main species affected, however, other species such as sheep, goats, pigs and so forth are also susceptible [1]. Pyrexia, lethargy, lameness and extra salivation with vesicles and erosions in the mouth, on the teats and on the feet are the typical clinical signs of FMD [2, 3]. The causative virus can be transmitted from infected animals to other susceptible animals by direct contact, fomites, animal products, contaminated surfaces and through the air [4]. Although the mortality rate of FMD is less than 5%, the importance of the disease is due to its negative effects on the marketing of animals and animal products, impacting on the national economy and return to trade of animals and the livestock industry [5].

The FMD virus belongs to the genus Aphthovirus, family Picornaviridae and has seven different serotypes including, A,C,O,SAT1,SAT2,SAT3 and Asia1 [6]. Three of the seven FMD serotypes (A,O and Asia1) have been circulating in Iran since 2011 [7–9].

Control of FMD in endemic regions such as Iran is mainly focused on mass vaccination of all susceptible livestock with a tetravalent vaccine [10] identification and testing of animals, establishment of protection and surveillance zones and enforcement of quarantine and biosecurity (Iranian Veterinary Organization). Despite the implementation of the national FMD control program, Iran is still faced with a number of challenges with respect to FMD status in that several outbreaks are still reported nationally. One of the worse affected areas in Iran is the province of Khorasan Razavi where 119 outbreaks of clinical FMD declared in 2012, 55 outbreaks in 2013 and 123 outbreaks in 2014 (GISVET database, IVO). This resulted in an annual FMD incidence rate between 0.73 and 4.63% of epidemiological-units. According to these statistics, we can claim that FMD virus transmission may have gone unnoticed in this endemic situation and with variable preventive measures. FMD vaccination is administered routinely by IVO in the province using a non-purified tetravalent vaccine containing serotypes A, Asia 1 and two strains of serotype O, which were produced locally in Razi institute (Tehran, Iran). Since 2011, current vaccination protocol by the IVO declares that vaccination needs to be applied three times a year for cattle in Khorasan Razavi as well as the country.

Usage of real-time case-control studies is uncommon in animal disease outbreaks and the majority of recent knowledge on FMD transmission originated from experimental studies and simulation modelling rather than field data [11, 12]. Previous studies investigating risk factors for FMD recurrence have identified various risk factors such as low effectiveness of the used vaccine [13, 14], long periods between vaccination and infection [14], proximity to borders [15], exposure to infected wild animals [16, 17], movement of infected animals [18–20] and lack of suitable biosecurity [19, 21, 22]. However, for the province of Khorasan Razavi very little is known about the determinants associated with the recurrence of FMD outbreaks in the region; this will help to distinguish why certain herds or farms in the region are at a higher risk of having FMD than others [23, 24].

This study is the first to investigate risk factors for the recurrence of FMD outbreaks in northeastern Iran. In this study we aimed to analyse FMD outbreak data for a three-year period (2012–2014) in the Khorasan Razavi province (northeastern Iran) to quantify the risk factors for FMD infection in order to inform evidence-based disease control recommendations to prevent future outbreaks.

## Methods

### Study population

**Fig. 1:**
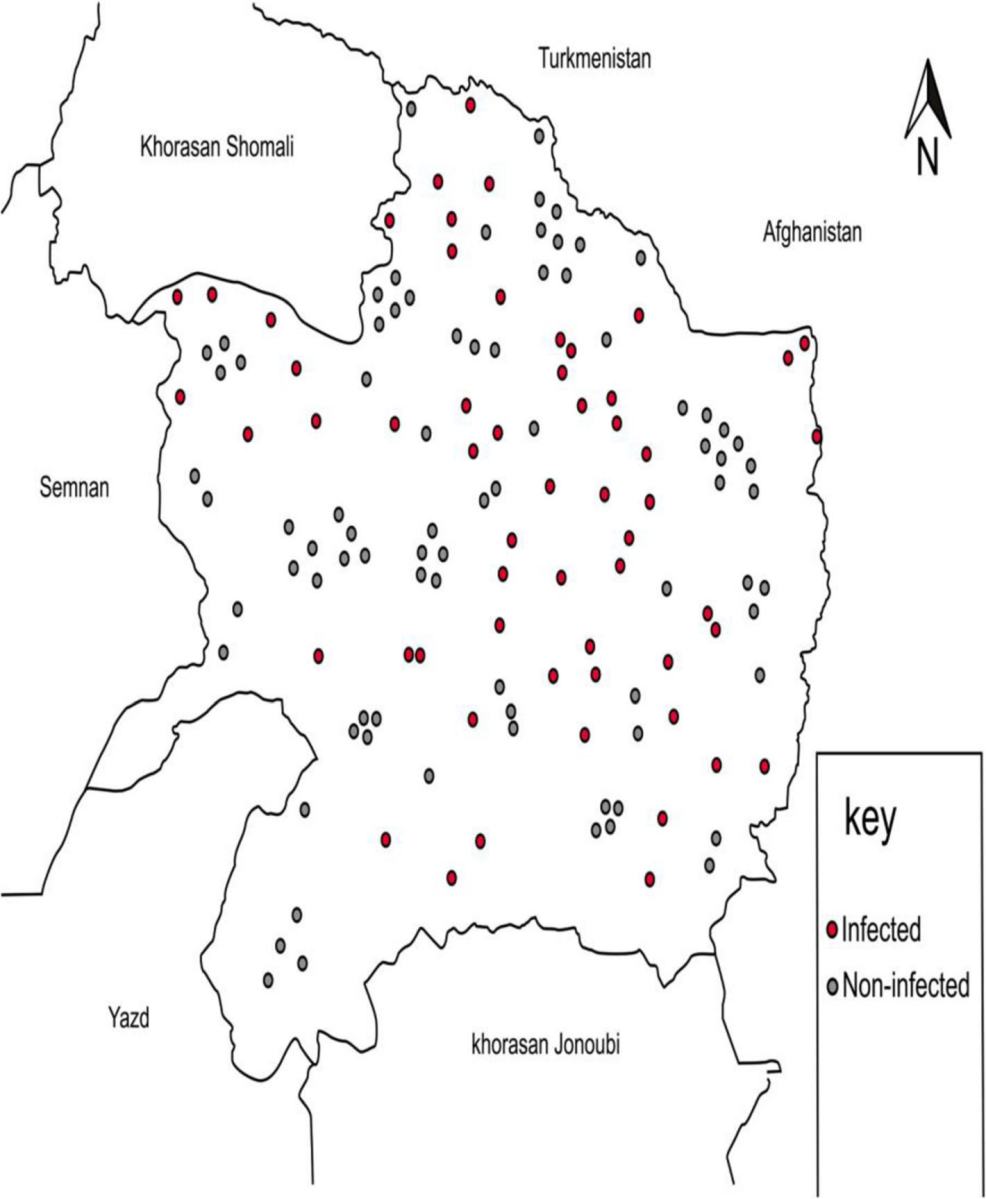
Map of Khorasan Razavi, northeastern of Iran. The infected farms are displayed by red dots and non-infected farms are showed by grey dots. The vaccinated areas included both infected and non-infected farms

To identify risk factors for recurrent FMD outbreaks between 2012 and 2014 in the Khorasan Razavi province, a case-control study was designed targeting all 138 farms (including 49,366 cattle) in the province. However, 11 farms declined to participate. A total of 127 farms (GISVET database, Iranian Veterinary Organization) were included in the investigation. The investigations were performed as part of the control measures applied by the Iranian Veterinary Organization in order to prevent further FMD outbreaks and were carried out based on the ethical standards agreed by the IVO for outbreak investigations. Khorasan Razavi province, which is a 119,000 km^2^ area close to the borders with Afghanistan and Turkmenistan, has one of the highest population density of cattle farms in Iran (Fig. 1). In 2014, there were an estimated 300,000 cattle and 7.4 million small ruminants in Khorasan Razavi province, which 80% of livestock accommodated in epidemiological-units including villages, commercial dairy herds and commercial mixed herds (combination of sheep, goats, beef and or dairy). The remaining part of livestock was distributed in nomadic herds. Holstein Friesians are the breed of dairy cattle in Khorasan Razavi province.

### Case definition for case and control farms

Classification of case farms was based on the appearance of clinical signs of FMD as observed by the herdsmen and the attending veterinarian in one or more animals and laboratory confirmation by polymerase chain reaction (PCR) by the IVO. Control farms were defined as those which the herdsmen and the attending veterinarian did not observe clinical signs compatible with FMD and a negative laboratory result by PCR. As a result, out of 127 farms a total of 46 case farms and 81 control farms were included in the study. The case and control farms were matched based on herd size; 35 case farms were matched with two control farms while the remaining 11 case farms were matched with one control farm. That is because we could not find suitable control farms for those 11 case farms based on herd size.

### Data collection

Risk factor data were collected for each case and control farm by means of a questionnaire composed of 13 questions on particular risk factors associated with FMD transmission which were organized into 4 sections: 1) general farm information, 2) movement of people, 3) movement of vehicles and 4) general farm management. Questions included in the risk factor questionnaire were designed based on the potential risk factors recognized by IVO in previous FMD outbreaks in Iran [unpublished observations, Mohamad Rashtibaf]. We evaluated the hygiene status of the farms by asking specific questions in the general farm management section in the questionnaire to consider the implementation of quarantine (i.e. separating infected animals from others, restriction movements of staff, veterinarians and vehicles) and biosecurity guidelines (i.e. presence of main gate, physical barrier to livestock area, wheel wash, boot dips, farm-specific clothing, clothes-changing area and so forth).

Data were collected from case and control farms by personnel from the province animal disease department and veterinarians of the Veterinary Council of the province by visiting each farm and interviewing the farm manager. Incomplete information was followed up by subsequent telephone interviews of the farm manager.

### Statistical analyses

The statistical analysis was conducted in two phases: firstly, all variables were examined by univariable analysis using a chi-squared or Fisher’s exact test; variables with *p*-value ≤0.15 in the univariable analysis were considered for the multivariable model [25]. Correlation between candidate variables for multivariable analysis were tested using the phi coefficient. Phi coefficient is a measure of association between two binary variables and is applicable to categorical variables. Among variables, those variables that were highly correlated (phi coefficient > 0.4), were selected for the multivariable analysis.

Secondly, following univariable variable screening, a conditional multivariable logistic regression model was built. To select the final multivariable model, a backward stepwise elimination approach was applied using *p* < 0.05 for variable retention. Confounding and effect modification between variables in the final model were evaluated. For the presence of confounding we evaluated the impact of removing a non-significant variable on the measure of disease association of other variables; if the odds ratio of a variable changed by 10%, after removal of a non-significant variable, we considered the latter as a confounder and retained in the multivariable model. For effect modification, we used − 2 log likelihood test from logistic regression to test the statistical significant of potential effect modifiers and calculate the estimators of exposure-disease association according to the level of significant effect modifiers. All statistical analyses were performed using SPSS version 17.0 (SPSS Inc., Chicago, IL, USA).

## Results

### Risk factors for FMD emergence

Results of univariable analyses on cattle farms in this area are shown in Table 1. Among 13 variables, a total of six explanatory variables (*p* < 0.15) were selected for the multivariable analysis: ‘hygienic status of the farm’, ‘vaccination’, ‘transportation of livestock’, ‘feed transport vehicles visited the farm’, ‘inhibition of livestock dealers’ entry in the farm’, and ‘the number of people involved in the livestock market’ as protective factors (Table 2). The final model was checked for goodness-of-fit using Hosmer-Lemeshow statistics [26]. Based on results of multivariable logistic regression, four factors, including hygienic status of the farm (OR = 11.83; CI = 3.375–41.43), FMD full course vaccination (OR = 0.06; CI = 0.005–0.684), transportation of livestock (OR = 0.40; CI = 0.163–0.981) and inhibition of livestock dealers’ entry into the farm (OR = 0.362; CI = 0.12–1.093) were identified as important factors influencing the occurrence of FMD.

**Table 1:** Results of univariable analysis for risk factors associated with FMD outbreaks

Variable	Category	Frequency		Odds ratio	%95 Confidence interval	*P* value
		Case	Control			
Type of livestock units	Traditional	38	69	0.826	0.311–2.197	0.702
	Dairy cattle farms	8	12			
Hygienic status of the farm	Suitable	19	8	6.421	2.517–16.383	< 0.001
	Not suitable	27	27			
Vaccination (every 4 months)	Yes	29	75	0.203	0.25–1.676	0.098
	No	17	6			
Rappel Vaccination	Yes	31	52	0.868	0.403–1.866	0.435
	No	15	29			
Transportation of livestock	Yes	29	26	0.227	0.130–0.592	0.001
	No	17	55			
Feed transport vehicles visited The farm	Yes	26	35	0.0585	0.282–1.215	0.149
	No	20	46			
Entrance of manure unloading vehicles	Yes	21	41	0.800	0.359–1.782	0.585
	No	32	60			
Entrance of Vehicles carrying Milk	Yes	30	50	0.860	0.405–1.829	0.695
	No	16	31			
Entrance of vaccinator groups	Yes	28	40	0.627	0.301–1.308	0.212
	No	18	41			
Entrance of artificial insemination groups	Yes	22	36	0.873	0.422–1.803	0.713
	No	24	45			
Inhibition of livestock dealers’ entry into the farm	Yes	27	23	0.279	0.130–0.597	0.001
	No	19	58			
Farm owners associated with livestock market	Yes	37	43	0.275	0.118–0.643	0.002
	No	9	38			
People associated with milk collection, artificial insemination and manure collection	Yes	38	69	1.211	0.455–3.22	0.702
	No	8	12

**Table 2:** Results of conditional multivariable analysis for risk factors associated with FMD outbreaks in Khorasan Razavi, Iran

Variable	Odds ratio	%95 Confidence interval	*P* value
Hygiene status of the farm	11.826	3.375–41. 434	< 0.001
Vaccination (every 4 months)	0.060	0.005–0.684	0.023
Transportation of livestock	0.400	0.163–0.981	0.045
Inhibition of livestock dealers’ entry into the farm	0.310	0.118–1.093	0.017

## Discussion

This study is one of the few studies on FMD outbreaks in Iran and the first one in northeastern Iran which evaluates risk factors for FMD infection. Apart from our study, there is only one study on FMD risk factors in northwestern Iran (West Azerbaijan) which is a serological cross-sectional study, and investigates prevalence of antibodies to non-structural proteins in young cattle showing FMD infection to assess potential risk factors for FMD outbreaks [19]. FMD outbreaks still occur annually in Iran despite the application of FMD control measures including, routine vaccination of livestock (i.e. cattle and small ruminants), the usage of emergency vaccination and post-movement quarantine based on the IVO protocol. In endemic countries, FMD outbreaks are frequently underreported in regard to level of economic and political development of the country [27]. This case-control study was carried out in order to investigate risk factors associated with the recurrence of FMD outbreaks in cattle farms located in the Khorasan Razavi province (northeastern Iran), an area in Iran heavily impacted by FMD outbreaks. The results of the current study which analysed data from 46 case and 81 control farms within the Khorasan Razavi province advance the knowledge base yielded by other field epidemiological investigations, and establish principles to inform evidence-based farm-level surveillance and control operations in the future.

Our results demonstrate that the hygiene status of the farms’ play an important role in FMD transmission in that the odds of lack of biosecurity in FMD-infected farms was 11 times greater than in non-infected farms. This finding is in line with recent evidence from other nearby countries. For example, an outbreak in four regions of Bhutan in 2009, it was considered that raising awareness of farmers is required to implement simple biosecurity procedures in FMD endemic villages in order to reduce the spread of the virus to other areas [22]. Recent outbreaks in West Azerbaijan (northwestern Iran) indicated that FMD prevention could not be achieved by vaccination alone in the endemic situations such as Iran [19]. Therefore, additional control measures like strict application of biosecurity and quarantine measures through monitoring professionals (veterinary practitioners, vaccinators, milk-collectors, inseminators) are required [19].

The most important factor in the long-range geographical spread of FMD in endemic areas is the movement of infected livestock through traditional or informal networks of livestock trade [18, 20, 28, 29]. In our study we demonstrated that ‘transportation of livestock’ is a crucial risk factor for FMD spread (OR = 0.40, CI = 16.3–981). Cattle are transported to different regions of Iran by dealers and herdsmen without any pre-movement testing; hence, the FMDV would be transmitted from infected cattle to the other susceptible livestock easily. In a similar study which was conducted in West Azerbaijan (northwestern Iran), more than 60% of herdsmen claimed that they traded livestock locally and distantly; moreover, this common trade is a social pass time for farmers throughout Iran which increases the risk of FMDV transmission. Therefore, limitation of animals’ movement has become the first priority of the veterinary services and stakeholders in Iran [19]. In fact, the IVO has recruited some veterinarians in the livestock markets in order to prevent excessive livestock movements.

Another risk factor influencing the occurrence of FMD in Iran is the movement of people strange to the farm, particularly those that have frequent contact with livestock such as dealer, traders, and middle men [29]. Dealers are people who trade animals and move regularly between farms and markets without any permission all over Iran (i.e. Khorasan Razavi province) and producers commonly move cattle from one market to another in search of better prices. Dealers transfer animals between farms and markets and can introduce the FMDV through infected animals and fomites [30]. In this situation, animals are kept for at least 1 to 3 days in the markets which result in the transmission of FMDV from infected to susceptible livestock.

According to the FMD vaccination protocol of Razi Institute (Tehran, Iran), cattle should be vaccinated three times a year. However, the results of our study in Khorasan Razavi province, indicate that some cattle in our study population had only been vaccinated once or twice at most. Therefore, a long period between vaccination and infection occur for herds that are not vaccinated according to the vaccination protocol [19]. Indeed our results confirm that the odds of lack of compliance in FMD vaccination (every 4 months) was greater in FMD-infected farms compared to non-infected farms suggesting that reducing the susceptibility of farms through full compliance with FMD vaccination is an important mitigating factor for the recurrence of outbreaks in the region. This finding could be partly explained by the fact that a few epi-units effectively received three annual vaccine rounds prior to the study. A possible explanation for this is the limited production capacity of vaccines in some years [19]. Previous studies indicate that timing and number of vaccine rounds are an important factor against FMD outbreaks [14]. During a FMD outbreak in Israel, a study was carried out on beef farms and illustrated a time period more than 6 months between adult vaccination and FMDV infection resulted in low protective effectiveness of vaccine and those farms were affected by FMD virus when the outbreak occurred [31].

The findings of this study need to be interpreted in light of a few limitations. First, it is known that case-control studies are based on questionnaires. To minimize the failures through collecting data by questionnaires in our study, the obtained data were validated by paper-based records and field veterinarians as well as GISVET database of Iranian Veterinary Organization. Second, case-control studies include detection of infection origin, identification of risk factors and aid case findings and control during an outbreak; however, such studies present no information about cause and effect [32–35].

## Conclusions

Our findings enabled an insight into risk factors for the recurrence of FMD outbreaks in Iran and have generated potential recommendations for farmers. The identified risk factors from this study may contribute to the risk-based strategy plan in Iran, as part of the Progressive Control Pathway for FMD control. Continuous efforts including further follow-up studies should be undertaken to evaluate the efficacy of strategies that aim to tackle the risk factors identified in this study.

## Abbreviations

FMD: Foot-and-mouth disease
GIS: Geographical Information System
IVO: Iranian Veterinary Organization
PCR: Polymerase Chain Reaction
